# Supplementary material for: Exploring the path to optimal diabetes care by unravelling the contextual factors affecting access, utilisation, and quality of primary health care in West Africa: A scoping review protocol
Source: PLoS One. 2024 May 20;19(5):e0294917. doi: 10.1371/journal.pone.0294917 (PMC11104679; doi:10.1371/journal.pone.0294917)
Supplement: S1 Table — (DOCX) [file pone.0294917.s002.docx]

**S1 Table – Key Words Combination Chart**

| **Population** | **Concept** | **Context** | **Outcome** |
| --- | --- | --- | --- |
| Adults OR Human beings in the age group of 18 years and above  AND  Diabetes OR Diabetes Mellitus OR Diabetic condition OR High blood sugar OR Glucose intolerance OR Metabolic disorder OR Sugar disease OR Hyperglycemia OR Insulin Resistance OR Insulin Deficiency | - Access OR Availability OR Admission OR Reachability OR Approach - Utilisation OR Usage OR Application OR Utilization OR Implementation OR Use OR Attendance OR Uptake - Quality OR Excellence OR Standard OR Efficiency OR Effectiveness OR High level - Factors OR Influencing elements OR Barriers OR Facilitators OR Determinants OR Enablers | West Africa OR Sub-Saharan Africa OR Western African region OR Countries in West Africa (e.g., Benin, Burkina Faso, Cape Verde, Gambia, Ghana, Guinea, Guinea-Bissau, Ivory Coast, Liberia, Mali, Mauritania, Niger, Nigeria, Senegal, Sierra Leone, Togo) | Primary Health Care OR Primary care services OR Community health services OR Basic healthcare OR Initial medical care OR Primary medical assistance |
