# Supplementary material for: Exploring the path to optimal diabetes care by unravelling the contextual factors affecting access, utilisation, and quality of primary health care in West Africa: A scoping review protocol
Source: PLoS One. 2024 May 20;19(5):e0294917. doi: 10.1371/journal.pone.0294917 (PMC11104679; doi:10.1371/journal.pone.0294917)
Supplement: S2 Table — (DOCX) [file pone.0294917.s003.docx]

**S2 Table – Search Terms for PubMed**

| 1 | "Diabetes Mellitus, Type 2"[Mesh]) OR "Diabetes Mellitus"[Mesh] OR "Diabetes Mellitus, Type 1"[Mesh] OR “Diabetes mellitus*”[tw] OR Diabet*[tw] OR “Type 1 diabetes*”[tw] OR “Type 2 diabetes*”[tw] OR Hyperglycemia[tw] OR “High blood sugar*”[tw] OR “Elevated blood glucose*”[tw] OR “Insulin resistance*”[tw] OR “Insulin deficiency*”[tw] |
| --- | --- |
| 2 | "Primary Health Care"[Mesh] OR “Primary health care*”[tw] OR “Primary care*”[tw] OR “primary health servic*”[tw] OR “Community health servic*” OR “Basic healthcare”[tw] OR “Initial medical care”[tw] OR “Primary medical assistance”[tw] |
| 3 | "Access to Primary Care"[Mesh]) OR "Health Services Accessibility"[Mesh] OR Access[tw] OR Availability[tw] OR Reach*[tw] OR Admission[tw] |
| 4 | Utilisation[tw] OR Uptake[tw] OR Usage[tw] OR Utilization[tw] OR Engagement[tw] OR Attendance[tw] OR Utility[tw] |
| 5 | "Quality of Health Care"[Mesh] OR Quality[tw] OR Standard[tw] OR Effectiveness[tw] OR Excellence[tw] OR Efficiency[tw] OR “High level*”[tw] |
| 6 | “Factors” OR “Barriers” OR “Challenges” OR “Enablers” OR “Facilitators” OR “Determinants” OR “influences” |
| 7 | "Africa, Western"[Mesh] |
| Combine | *#1 AND #2 AND #3 AND #4 AND #5 AND #6 AND #7* |
