# Supplementary material for: Exploring the path to optimal diabetes care by unravelling the contextual factors affecting access, utilisation, and quality of primary health care in West Africa: A scoping review protocol
Source: PLoS One. 2024 May 20;19(5):e0294917. doi: 10.1371/journal.pone.0294917 (PMC11104679; doi:10.1371/journal.pone.0294917)
Supplement: Attachment — Submitted filename: PONE-D-23-22733.pdf [file pone.0294917.s007.pdf]

PLOS ONE

Exploring the path to optimal diabetes care by unravelling the contextual factors affecting access, utilisation, and quality of primary health care in West Africa: a scoping review protocol

--Manuscript Draft--

**Manuscript Number:**	PONE-D-23-22733
**Article Type:**	Study Protocol
**Full Title:**	Exploring the path to optimal diabetes care by unravelling the contextual factors affecting access, utilisation, and quality of primary health care in West Africa: a scoping review protocol
**Short Title:**	Factors influencing diabetes care in West Africa: A scoping review protocol
**Corresponding Author:**	Abdul-Basit Abdul-Samed, M.D<br>Ghana College of Physicians and Surgeons<br>Accra, Greater Accra GHANA
**Keywords:**	diabetes; diabetic; access; Availability; Utilisation; utilization; usage; Uptake; Quality; standard; factors; determinants; barriers; facilitators; Primary health care; primary care
**Abstract:**	Background<br>The increasing prevalence of diabetes in West Africa poses a pressing public health challenge. Primary health care (PHC) has been identified as a crucial tool to reduce this menace, making it essential to comprehend the factors that affect access, utilisation and quality of PHC for diabetes in West Africa. This will allow the development of successful interventions to improve patient outcomes. Our objective with this scoping review protocol is to explore the literature to reveal the contextual factors that affect primary health care for diabetes in West Africa.<br>Methods and Analysis<br>Using the established methodology by Arksey and O'Malley, this scoping review will undergo six stages: 1. Formulating the research question, 2. Identifying relevant studies, 3. Selecting eligible literature, 4. Charting the data, 5. Collating, summarising and reporting the results, and 6. Peer consultation for feedback and validation. This protocol will adopt the Preferred Reporting Items for Systematic Reviews and Meta-Analysis Extension for Scoping Review (PRISMA-ScR) guidelines to ensure methodological rigour. We will search four electronic databases (PubMed, Google Scholar, African Journals Online, and CAIRN Info) and search through grey literature sources to thoroughly explore the topic. The identified articles will undergo thorough screening by two independent reviewers based on the pre-established inclusion and exclusion criteria. In case of any discrepancies, a third reviewer will be consulted for resolution through discussion. We will collect data from studies using a standardised data extraction form that covers study characteristics, population demographics, study methods, and factors affecting access, utilisation, and quality of primary healthcare for diabetes. The study will identify key themes and sub-themes related to primary healthcare access, utilisation, and quality. We will then analyse and summarise the data using a narrative synthesis approach.<br>Results<br>It is expected that the findings and conclusive report will be finished and sent to a peer-reviewed publication within six months.<br>Conclusion<br>This review protocol aims to systematically examine and assess the factors that impact the access, utilisation, and standard of primary healthcare services for diabetes in West Africa.
**Order of Authors:**	Abdul-Basit Abdul-Samed
	Ellen Barnie Peprah
	Tolib Mirzoev
	Irene Agyepong

Powered by Editorial Manager® and ProduXion Manager® from Aries Systems Corporation

	Henry Lawson
	Yasmine Jahan
	Veronika Reichenberger
	Edward Antwi
**Additional Information:**	
**Question**	**Response**
**Financial Disclosure**<br><br>Enter a financial disclosure statement that describes the sources of funding for the work included in this submission. Review the submission guidelines for detailed requirements. View published research articles from *PLOS ONE* for specific examples.<br><br>This statement is required for submission and **will appear in the published article** if the submission is accepted. Please make sure it is accurate.<br><br>**Unfunded studies**<br>Enter: *The author(s) received no specific funding for this work.*<br><br>**Funded studies**<br>Enter a statement with the following details:<br>• Initials of the authors who received each award<br>• Grant numbers awarded to each author<br>• The full name of each funder<br>• URL of each funder website<br>• Have or will the sponsors or funders play any role in the study design, data collection (when applicable) and analysis, decision to publish, or preparation of the manuscript?<br>• **NO** - Include this sentence at the end of your statement: *The funders did not and will not have a role in study design, data collection and analysis, decision to publish, or preparation of the manuscript.*<br>• **YES** - Specify the role(s) played.<br><br>* typeset	I.A. and T.M. received funding from the National Institute for Health Research (NIHR) Global Health Research Centre (grant number NIHR203246). The funder for this grant is the NIHR Global Health Research Centre on Strengthening of Capacity for NCD Control in West Africa (Stop-NCD) (https://nihr.ac.uk/). The focus of this research is to strengthen the capacity for NCD control in West Africa.
**Competing Interests**<br><br>Use the instructions below to enter a competing interest statement for this submission. On behalf of all authors, disclose any competing interests that	We declare no competing interests.

Powered by Editorial Manager® and ProduXion Manager® from Aries Systems Corporation

could be perceived to bias this work—acknowledging all financial support and any other relevant financial or non-financial competing interests.<br><br>This statement is required for submission and **will appear in the published article** if the submission is accepted. Please make sure it is accurate and that any funding sources listed in your Funding Information later in the submission form are also declared in your Financial Disclosure statement.<br><br>View published research articles from *PLOS ONE* for specific examples.<br><br>**NO authors have competing interests**<br><br>Enter: *The authors have declared that no competing interests exist.*<br><br>**Authors with competing interests**<br><br>Enter competing interest details beginning with this statement:<br><br>*I have read the journal's policy and the authors of this manuscript have the following competing interests: [insert competing interests here]*<br><br>* typeset	
**Ethics Statement**<br><br>Enter an ethics statement for this submission. This statement is required if the study involved:<br><br>• Human participants<br>• Human specimens or tissue<br>• Vertebrate animals or cephalopods<br>• Vertebrate embryos or tissues<br>• Field research<br><br>Write "N/A" if the submission does not require an ethics statement.	N/A

General guidance is provided below. Consult the submission guidelines for detailed instructions. **Make sure that all information entered here is included in the Methods section of the manuscript.**<br><br>**Format for specific study types**<br><br>**Human Subject Research (involving human participants and/or tissue)**<br>• Give the name of the institutional review board or ethics committee that approved the study<br>• Include the approval number and/or a statement indicating approval of this research<br>• Indicate the form of consent obtained (written/oral) or the reason that consent was not obtained (e.g. the data were analyzed anonymously)<br><br>**Animal Research (involving vertebrate animals, embryos or tissues)**<br>• Provide the name of the Institutional Animal Care and Use Committee (IACUC) or other relevant ethics board that reviewed the study protocol, and indicate whether they approved this research or granted a formal waiver of ethical approval<br>• Include an approval number if one was obtained<br>• If the study involved *non-human primates*, add *additional details* about animal welfare and steps taken to ameliorate suffering<br>• If anesthesia, euthanasia, or any kind of animal sacrifice is part of the study, include briefly which substances and/or methods were applied<br><br>**Field Research**<br><br>Include the following details if this study involves the collection of plant, animal, or other materials from a natural setting:<br>• Field permit number<br>• Name of the institution or relevant body that granted permission	
**Data Availability**<br><br>Authors are required to make the data	N/A - No results are reported

underlying their research findings fully available, without restriction.<br><br>If the manuscript reports pilot or preliminary results, the data underlying those results must be made available at the time of publication. A Data Availability Statement describing where the data can be found is required at submission. Your answers to this question constitute the Data Availability Statement and will be published in the article, if accepted. When legal or ethical requirements prohibit public data sharing, authors must indicate how researchers may obtain access to the data. See the PLOS Data Policy and FAQ for detailed information.<br><br>**Important:** Stating 'data available on request from the author' is not sufficient. If your data are only available upon request, select 'No' for the first question and explain your situation in the text box.<br><br>For protocols without pilot or preliminary data, authors are strongly encouraged to state how they plan to share research data from their study when it is completed or published.<br><br>If your study includes pilot data, will they be made fully available without restriction?	
**Describe where the data may be found in full sentences. If you are copying our sample text, replace any instances of XXX with the appropriate details.**<br><br>• If **no pilot data are reported** in the manuscript, please enter the following: *No datasets were generated or analysed during the current study. All relevant data from this study will be made available upon study completion.*<br>• If the manuscript reports pilot data **held in a public repository**, include URLs,	No datasets were generated or analysed during the current study. All relevant data from this study will be made available upon study completion.

accession numbers or DOIs. If this information will only be available after acceptance, indicate this by ticking the box below. For example: *All XXX files are available from the XXX database (accession number(s) XXX, XXX.).*<br>• If the manuscript reports pilot data contained **within the manuscript and/or Supporting Information files**, enter the following:<br>*All relevant data are within the manuscript and its Supporting Information files.*<br>• If neither of these applies but you are able to provide **details of access elsewhere**, with or without limitations, please do so. For example:<br><br>*Data cannot be shared publicly because of [XXX]. Data are available from the XXX Institutional Data Access / Ethics Committee (contact via XXX) for researchers who meet the criteria for access to confidential data.*<br><br>*The data underlying the results presented in the study are available from (include the name of the third party and contact information or URL).*<br>• This text is appropriate if the data are owned by a third party and authors do not have permission to share the data.<br><br>For study protocols with data management or sharing plans, authors are encouraged to briefly describe in their Data Availability Statement how data they generate will be made accessible when the study is completed. For example:<br>• Deidentified research data will be made publicly available when the study is completed and published.<br><br>* typeset	
Additional data availability information:	

Manuscript
Click here to access/download;Manuscript;Manuscript - Diabetes.docx

Exploring the path to optimal diabetes care by unravelling the contextual factors affecting access, utilisation, and quality of primary health care in West Africa: a scoping review protocol

Abdul-Basit Abdul-Samed*[1], Ellen Barnie Peprah[¶1], Tolib Mirzoev[¶2], Irene Agyepong[¶1], Henry Lawson[&1] ,Yasmine Jahan[&2], Veronika Reichenberger[&2], Edward Antwi[¶3]

[1]Ghana College of Physicians and Surgeons, Accra, Ghana

[2]London School of Hygiene and Tropical Medicine, London, United Kingdom

[3]Ghana Health Service, Accra, Ghana

*Corresponding Author

abdulbasitgunu@gmail.com

[¶]These authors contributed equally to this work

[&]These authors also contributed equally to this work

Funding

This work was supported by the National Institute for Health Research (NIHR) Global Health Research Centre on Strengthening of Capacity for NCD Control in West Africa (Stop-NCD) under grant number NHIR203246. The funders had no role in study design, data collection and analysis, decision to publish, or preparation of the manuscript.

Conflict of Interest

We declare no conflict of interest for this proposed study.

Data Availability

As this is a protocol for a scoping review, there are no primary data sets associated with this paper. The scoping review will synthesise and analyse data from published studies that meet the eligibility criteria outlined in the protocol.

All studies included in the final scoping review will be cited in a reference list. The full search strategy for databases will be made available in the final manuscript. Charting of extracted data from eligible studies will be included in tabular form in the published scoping review results. Any additional materials or data required to reproduce the scoping review analysis will be provided upon reasonable request to the corresponding author.

Abstract

Background

The increasing prevalence of diabetes in West Africa poses a pressing public health challenge. Diabetes is ambulatory care sensitive in that access to good primary health care is expected to reduce complications, morbidity and mortality. Primary health care (PHC) has been identified as a crucial tool to reduce this menace, making it essential to comprehend the factors that affect access, utilisation and quality of PHC for diabetes in West Africa. This will allow the development of successful interventions to improve patient outcomes. Our objective with this scoping review protocol is to explore the literature to reveal the contextual factors that affect primary health care for diabetes in West Africa.

Methods and Analysis:

Using the established methodology by Arksey and O'Malley, this scoping review will undergo six stages: 1. Formulating the research question, 2. Identifying relevant studies, 3. Selecting eligible literature, 4. Charting the data, 5. Collating, summarising and reporting the results, and 6. Peer consultation for feedback and validation. This protocol will adopt the Preferred Reporting Items for Systematic Reviews and Meta-Analysis Extension for Scoping Review (PRISMA-ScR) guidelines to ensure methodological rigour. We will search four electronic databases (PubMed, Google Scholar, African Journals Online, and CAIRN Info) and search through grey literature sources to thoroughly explore the topic. The identified articles will undergo thorough screening by two independent reviewers based on the pre-established inclusion and exclusion criteria. In case of any discrepancies, a third reviewer will be consulted for resolution through discussion. We will collect data from studies using a standardised data extraction form that covers study characteristics, population demographics, study methods, and factors affecting access, utilisation, and quality of primary healthcare for diabetes. The study will identify key themes and sub-

themes related to primary healthcare access, utilisation, and quality. We will then analyse and summarise the data using a narrative synthesis approach.

Results

It is expected that the findings and conclusive report will be finished and sent to a peer-reviewed publication within six months.

Conclusion

This review protocol aims to systematically examine and assess the factors that impact the access, utilisation, and standard of primary healthcare services for diabetes in West Africa.

Ethical Approval

Ethical approval nor participant consent is not required.

Key Words

diabetes, diabetic, access, availability, utilisation, usage, uptake, quality, standard, factors, determinants, barriers, facilitators, primary health care, primary care

Introduction

Sub-Saharan Africa (SSA) is currently grappling with the double burden of disease – communicable and non-communicable - which is impacting the lives of millions across the continent (1). Diabetes, a non-communicable disease, is a health condition where the body either does not produce enough insulin or cannot use it effectively (2). Insulin is a hormone produced in the body to regulate blood sugar levels. It functions as a key, providing the means for glucose to enter our body cells and be utilised as a source of energy. In diabetes, absolute or relative insulin deficiency, or both, leads to elevated blood sugar levels, which can cause various health problems (3).

Diabetes is a growing problem in SSA, and it is notably situated at the intersection of infectious and chronic disease processes and presents a significant threat to public health (4–6). Approximately 24 million individuals are living with diabetes in Africa, and by 2045, this figure is projected to grow by 129%(7). However, given the dearth of quality data and research on diabetes in SSA, prevalence estimates are uncertain as there is little evidence on the disease epidemiology(5,6). It is estimated that West Africa has a comparatively higher prevalence of diabetes compared to other African regions(8). The prevalence of diabetes in countries like Ghana, Burkina Faso and Niger has been estimated at 2% – 2.6%, 1.7% – 2.1%, and 5.6%, respectively, based on data from the International Diabetes Federation (IDF) and the World Bank (9,10).

The increasing burden of diabetes in SSA is reinforced by its nature, which puts young and older populations at risk. Among the identified types of diabetes – Type 1, Type 2, Gestational diabetes mellitus and specific types of diabetes due to other causes(11), Type 2 diabetes accounts for the most cases in the region. By estimates, approximately 90% of people living with diabetes in SSA have Type 2 diabetes. Additionally, in the Africa Region of IDF, around 52 million people (aged 20-79) are estimated to have Impaired Glucose Tolerance (IGT), significantly increasing their risk of developing Type 2 diabetes (9).

Given the diverse cultural fabric of SSA, the quality of diabetes care is impacted by unique challenges. Cultural beliefs, misconceptions, and stigma surrounding diabetes, among others, can hinder effective care, patient-provider relationships, and treatment adherence (12,13). Primary health care (PHC), which serves as the basic level of care for managing diseases, including diabetes, is pivotal in addressing these challenges and delivering comprehensive care. According to the WHO, PHC is widely recognised as the most inclusive, equitable, and cost-effective approach to achieving universal health coverage (14,15); however, many parts of the developing world, including SSA, face obstacles to access, utilisation, and quality of primary health care services. Various systemic and contextual factors influence healthcare delivery in these regions, resulting in disparities and gaps in diabetes care (16). Addressing these challenges is crucial to providing patient-centred care that is culturally sensitive and respects the individual's beliefs, ideas, concerns, expectations and preferences. This will consequently lead to improved health outcomes for people living with diabetes.

Access and Utilisation

Access to PHC services is a prerequisite for effectively managing diabetes, preventing complications, and improving patient outcomes. Despite this, access to healthcare in Sub-Saharan Africa is estimated to be 42.56%, highlighting the critical need to eliminate barriers that limit access to essential healthcare services (17). According to a review by Bresick et al., the state and performance of PHC in SSA need to be adequately measured, indicating a gap in the current research landscape (18).

Whereas "access to healthcare" can be described as the opportunity to fulfil healthcare needs, it encompasses various dimensions, including approachability, acceptability, affordability, availability and appropriateness (19). Across the various dimensions, many factors influence access to and utilisation of care in the developing world. These include inadequate healthcare infrastructure, limited resources, inadequate healthcare workforce, sociocultural beliefs and practices, and economic disparities (19,20).

In Lower and Middle-Income Countries (LMICs), including those in West Africa, geographic barriers play a pivotal role in the underutilisation of primary healthcare for diabetes. The literature suggests a significant unmet need for diabetes care in parts of SSA, indicating that health services utilisation may be suboptimal (21). Remote and underserved communities often lack easy access to healthcare facilities, resulting in delayed diagnoses, limited follow-up care, and compromised disease management (22). Additionally, cultural beliefs, traditional healing practices, and misconceptions surrounding diabetes can impact healthcare-seeking behaviour, leading to the underutilisation of orthodox medicine. The utilisation patterns of people living with Non-communicable Diseases (NCDs) are further influenced by various factors, including awareness or knowledge of diabetes, attitudes towards seeking care and availability of services. The burden of diabetes further compounds the strain on already overburdened healthcare systems, necessitating innovative solutions to promote the utilisation of available care.

Access to and utilisation of healthcare services are closely interconnected, and certain factors can impact both aspects simultaneously. While it may be challenging to separate these factors, it is essential to understand their distinct influences:

Financial Limitations: Accessing healthcare services can become challenging when financial resources are limited. High out-of-pocket expenses, absence of health insurance coverage, and the inability to afford transportation expenses can prevent people from accessing healthcare facilities. Furthermore, financial limitations can lead to underutilisation of services as some individuals may avoid seeking care or delay treatment due to cost concerns.(6)

Healthcare Infrastructure: Access and utilisation of quality healthcare can be hindered by insufficient infrastructure, such as poorly equipped facilities and inadequate medical supplies. When healthcare centres are understaffed or lack necessary equipment, individuals may face difficulties in obtaining proper care, which could result in reduced utilisation. These challenges may lead people to seek alternative sources of care or even forego treatment altogether.(6,22)

Healthcare Workforce Shortages: Insufficient staffing levels in healthcare, including a shortage of doctors and nurses, can negatively affect patient access and utilisation. When healthcare facilities are understaffed, patients may experience longer wait times and reduced access to necessary medical attention. This shortage can make it difficult for people to schedule timely appointments or receive comprehensive care, ultimately affecting their ability to utilise healthcare services. (23)

Sociocultural Beliefs and Practices: Social and cultural factors significantly impact people's access to and use of healthcare services. Cultural beliefs and norms may discourage individuals from seeking proper medical care, which results in limited access. Additionally, cultural practices and preferences may influence the utilisation of healthcare services, with some people opting for traditional healers or home remedies instead of professional medical care. (12)

Quality

The World Health Organization defines the delivery of quality care as the extent to which health services improve the chances of achieving desired health outcomes for both individuals and populations. Achieving this requires a comprehensive approach that considers various aspects of care. This ensures that PHC services are safe, efficient, effective, patient-centred, integrated, equitable, and timely (24). In SSA, several barriers present significant challenges to achieving and upholding the necessary standards of care (16,25). Various factors identified in the literature which influence the quality of care include resource limitations, health worker training and capacity building, adherence to clinical guidelines and patient-provider communication (5,22).

Managing diabetes is a continuous process that requires consistent care and support to prevent complications and maintain good health. To ensure effective management of the condition, it is essential

to identify and address the contextual factors that influence PHC for diabetes to help provide ongoing care and support, prevent complications, and promote overall well-being (26).

Rationale

In order to have an adequate grasp of the contextual factors that favour or impede efficient PHC for diabetes in West Africa, it is crucial to synthesise the existing evidence on contextual factors influencing primary care access, quality and utilisation. Additionally, while much research has emphasised diabetes prevalence and risk factors, it is also critical to understand contextual influences on access to and utilisation of PHC services, treatment outcomes, and quality of care for diabetes patients (6,27,28). Our scoping review protocol aims to address the pressing public health concern of diabetes in West Africa by understanding these contextual factors to provide valuable insights for policy-makers, healthcare providers, and researchers. Through identifying barriers, gaps, and successful strategies, this review can potentially contribute towards improving primary healthcare for diabetes, enhancing patient outcomes, promoting health equity, and ultimately helping reduce the burden of diabetes in West Africa

Objectives

1. To identify and synthesise existing literature on the contextual factors influencing access, utilisation, and quality of primary healthcare for diabetes in West Africa, and how and why they work.
2. To identify gaps in the current literature for further research to inform intervention to improve access, utilisation, and quality of primary healthcare for diabetes in West Africa.

Conceptual Framework for the Scoping Review

To guide our review we will draw on a conceptual framework to rigorously examine multi-level contextual factors shaping primary care services for diabetes patients in the region. Context refers to the.surroundings or mileau in which a particular event or phenomenon is being studied; context is increasingly recognised as a crucial factor shaping health and healthcare (27). For the purpose of this scoping review, context refers to the diverse physical, social, cultural, economic, political, and health systems environments that influence opportunities for optimal diabetes prevention and care. Contextual factors interact across macro, meso, and micro levels (28,29). Macro-level elements include cultural norms, health policies, and demographic changes. Meso-level factors consist of community resources, healthcare facilities, staffing, and medical supply chains. Micro-level factors encompass individual attitudes, knowledge, interpersonal relationships, and living conditions. These contextual factors interact to impact PHC access, utilisation, and quality in West Africa, acting as both barriers and enablers.

(**Fig 1 – Conceptual Framework for the scoping review**)

Methodology

A preliminary literature scan indicates little published evidence on PHC for diabetes in West Africa; a scoping review is an appropriate method for mapping out any available evidence in this area. The methodology of the protocol for the scoping review will adhere to the framework created by Arksey and O'Malley (30,31). The methodology will therefore include the following stages:

1. Formulating the research question
2. Identifying relevant literature
3. Study Selection
4. Charting the data

5. Collating, summarising and reporting the results
6. Peer consultation, feedback and validation

In order to conduct and report the scoping review in a well-organised manner, we ~~will be utilising~~ the recommended tools by the Joana Briggs Institute(32), namely the Population, Concept, and Context (PCC) framework and the PRISMA-ScR (Preferred Reporting Items for Systematic Reviews and Meta-Analyses Extension for Scoping Reviews) guidelines and checklist.

Stage 1 - Identifying the Review Question

Through an iterative process and extensive consultation with the research team, the research question for the review has been carefully identified as follows:

What contextual factors influence primary healthcare access, utilisation and quality for diabetes in West Africa, and how and why do they work and what are the gaps in the current literature?

Stage 2 - Identifying Relevant Studies

To identify the studies, keyword searches of academic databases ~~will be~~ conducted. A comprehensive search strategy ~~will be~~ developed to identify relevant literature from four selected electronic databases, i.e. PubMed, Google Scholar, African Journals Online (AJOL), and CAIRN INFO (for French papers). In addition, grey literature sources such as the World Health Organization (WHO) website, the NCD Alliance and Ministries of Health where possible. This ~~will be~~ done with the help of a librarian. The search strategy ~~will be~~ developed using a combination of keywords and alternative vocabulary terms derived from the key concepts of the study (**Table in S1 Table**). A complete search strategy, applied to PubMed (**Table in S2 Table**), ~~will be~~ subsequently developed and then adapted to other databases. The search results ~~will be~~ managed using reference manager software (for example, EndNote, Zotero).

Stage 3 - Study Selection

The Population, Concept and Context Study framework shown in *Table 1* will align the inclusion of studies. The framework will be used to guide title and abstract screening. The inclusion and exclusion criteria shown in *Table 2* will initially guide the review. The criteria will iteratively undergo further refinement as the reviewers gain a better understanding of the literature's scope.

Table 1- Population, Concept and Context Framework

PCC Element	Definition/ Keywords
**Population**	Adults (Age 18 and above) living with diabetes (Type 1or Type 2)
**Concept**	• Access<br>• Utilisation<br>• Quality<br>• Primary Health Care
**Context**	West Africa

A decision-making flowchart **(Figure in S4 Figure)** will aid the study selection process.

Table 2 - Inclusion and Exclusion Criteria

	**Inclusion**	**Exclusion**
**Year of publication**	No limit	N/A
**Language**	English, French	Other Languages
**Population**	Adults with diabetes	People with gestational diabetes
**Concept**	Access<br>Utilisation<br>Quality<br>PHC	N/A
**Context**	West Africa	Regions outside West Africa
**Study design**	• Qualitative, Quantitative (if any), Meta-analysis, Social Science Case studies/reports<br>• Multi-country studies which include West African countries	• Studies that do not contain information on factors affecting access, utilisation and quality of PHC<br>• Abstracts<br>• Commentaries<br>• Opinions<br>• Scoping or Systematic review protocols

NA – Not Applicable

The abstracts will subsequently be reviewed for any mention of factors that influence access, utilisation or quality of PHC in adults with diabetes in West Africa. Due to its convenience, reviewers will use Rayyan software to independently screen the articles based on the inclusion and exclusion criteria. After the

initial abstract review is completed, the full texts will be accessed for final screening and data extraction. To ensure the reliability and validity of the source selection process, two reviewers will independently perform source selection at both the title/abstract screening and full-text screening stages. Any disagreements among the reviewers will be resolved by consensus or by the decision of an added reviewer.

A pilot test will be undertaken on the process to ensure effective source selection by randomly selecting a 5% sample of titles/abstracts. The entire team will screen them according to the eligibility criteria and discuss any discrepancies. The selection process will continue until a 75% agreement or higher is reached. A record of the progress of the source-selection process will be maintained for all full-text articles retrieved, including details of included and excluded sources. Any excluded sources will have reasons clearly stated for why they were excluded.

Stage 4 - Charting the Data

We will use a data charting/extraction tool (*Table 3*) recommended by the Joana Brigg's Institute(33) to capture relevant information from each study.

Table 3- Data Extraction Tool

**Author and Date**	
**Title**	
**Aim**	
**Study Setting**	
**Study Population**	
**Sampling Method**	
**Study Design**	
**Data Collection Method**	
**Data Analysis**	
**Conclusion**	
**Outcome**	
**Most relevant Findings**	
**Comment**	

Stage 5 - Collating, Summarising and Reporting the Results

To ensure transparency and clarity in reporting findings, the review team will be utilising the PRISMA (Preferred Reporting Items for Systematic Reviews and Meta-Analyses) flow diagram (**Fig. 2**) along with the PRISMA-ScR checklist (**Table in S5 Table**) (34,35).

Fig. 2 – PRISMA Flowchart

In collating the results, the data extracted from the included studies will be organised and categorised based on the key themes and concepts: access, utilisation and quality. In order to gain a comprehensive understanding of the factors impacting access, utilisation, and quality of primary healthcare for diabetes in West Africa, the team will systematically review and identify patterns, trends, and variations across the studies. Data segments that address the research questions and objectives will be pooled and analysed. Once the results have been collated, they will be summarised clearly and concisely. Reporting the results will involve the creation of a comprehensive narrative that presents the synthesised findings in a logical and structured manner. The narrative will summarise the key findings, including the prevalence and distribution of the identified factors and any variations observed across different countries or regions within West Africa. The results will be discussed in light of the research questions and objectives, highlighting the implications for policy, practice, and future research.

Stage 6 – Peer Consultation for Feedback and Validation

As we work on the project, we will collaborate with subject matter specialists to gain their insights and receive feedback. In stage 2, a skilled librarian will assess the search strategy through a peer review process to ensure that it is comprehensive and relevant. In addition, we plan to utilise our network to engage more experts in the field so that all relevant discoveries are reported with precision and clarity. Throughout stages 3 to 5, we will maintain communication with these experts to gather their recommendations for further reading and to receive feedback on data extraction.

Discussion

This scoping review protocol seeks to methodically investigate and evaluate the elements that affect the accessibility, usage, and quality of primary healthcare for diabetes in West Africa. The goal is to carefully search and analyse relevant studies to gain insights into the factors that affect diabetes care in the region. A thorough search strategy to select the most informative studies will be employed. The findings of this scoping review will help to improve understanding of the current landscape of diabetes care in West Africa and provide valuable information to improve the delivery and outcomes of PHC for individuals with diabetes.

Strengths and Limitations

This review will follow an established framework and use a comprehensive systematic search to identify and analyse existing literature. Our search strategy is peer-reviewed, and we will consult with experts to guide our review and include relevant grey literature. The heterogeneity of the included studies may make it challenging to draw definitive findings or compare studies. It is worth noting that the scope of this evaluation is limited to studies conducted in West Africa, which may restrict the applicability of the findings to other regions or populations. Additionally, the review is confined to specific languages (English and French), which may also affect the findings.

Ethics and Dissemination

Ethical approval is not required as this scoping review involves the analysis of existing literature. The findings will be disseminated through various channels, including peer-reviewed publications, policy briefs, conferences, and active engagement with relevant stakeholders. The goal is to inform evidence-based healthcare practices, shape policy interventions, and foster meaningful improvements in diabetes care for diabetes in West Africa, informed by evidence of factors influencing PHC for diabetes.

Authors Contributions

The study design, conceptualisation, and original draft creation were by ABAS, under the supervision of EA and IA, who reviewed and approved the draft. EBP contributed to the review and editing of the manuscript. TM reviewed the draft and provided input and feedback on research objectives and research questions. HL, YJ and VR reviewed and edited the protocol. All authors approved the final version of the review manuscript.

Acknowledgement

This research was funded by the NIHR Global Health Research Centre for Non-Communicable Disease Control in West Africa using UK aid from the UK Government to support global health research. The views expressed in this publication are those of the author(s) and not necessarily those of the NIHR or the UK government.

Supporting Information

S1 Table – Key Words Combination Chart

S2 Table - Search Terms for PubMed

S3 Table - Sample search for PubMed

S4 Figure - Decision Flowchart

S5 Table – PRISMA-ScR Checklist

S6 Table – PRISMA-P Checklist

References

1. Kolling M, Winkley K, von Deden M. “For someone who’s rich, it’s not a problem”. Insights from Tanzania on diabetes health-seeking and medical pluralism among Dar es Salaam’s urban poor. Glob Health. 2010 May 4;6(1):8.

2. Centers for Disease Control and Prevention [Internet]. 2023 [cited 2023 Jun 19]. What is Diabetes? Available from: https://www.cdc.gov/diabetes/basics/diabetes.html

3. Diagnosis and Classification of Diabetes Mellitus | Diabetes Care | American Diabetes Association [Internet]. [cited 2023 Jul 4]. Available from: https://diabetesjournals.org/care/article/37/Supplement_1/S81/37753/Diagnosis-and-Classification-of-Diabetes-Mellitus

4. Magee MJ, Narayan KMV. Global confluence of infectious and non-communicable diseases — The case of type 2 diabetes. Prev Med. 2013 Sep 1;57(3):149–51.

5. Atun R, Davies JI, Gal EAM, Bärnighausen T, Beran D. Diabetes in sub-Saharan Africa: from clinical care to health policy. 2017;5.

6. Pastakia S, Pekny C, Manyara S, Fischer L. Diabetes in sub-Saharan Africa – from policy to practice to progress: targeting the existing gaps for future care for diabetes. Diabetes Metab Syndr Obes Targets Ther. 2017 Jun;Volume 10:247–63.

7. WHO | Regional Office for Africa [Internet]. 2023 [cited 2023 Mar 24]. African region tops world in undiagnosed diabetes: WHO analysis. Available from: https://www.afro.who.int/news/african-region-tops-world-undiagnosed-diabetes-who-analysis

8. Dessie G, Mulugeta H, Amare D, Negesse A, Wagnew F, Getaneh T, et al. A systematic analysis on prevalence and sub-regional distribution of undiagnosed diabetes mellitus among adults in African countries. J Diabetes Metab Disord. 2020 Sep 22;19(2):1931–41.

9. Members [Internet]. [cited 2023 Mar 24]. Available from: https://idf.org/our-network/regions-members/africa/members.html

10. Diabetes prevalence (% of population ages 20 to 79) - Niger | Data [Internet]. [cited 2023 Mar 24]. Available from: https://data.worldbank.org/indicator/SH.STA.DIAB.ZS?locations=NE

11. American Diabetes Association. 2. Classification and Diagnosis of Diabetes: Standards of Medical Care in Diabetes—2021. Diabetes Care. 2020 Dec 4;44(Supplement_1):S15–33.

12. Omodara DA, Gibson L, Bowpitt G. Exploring the impact of cultural beliefs in the self-management of type 2 diabetes among Black sub-Saharan Africans in the UK – a qualitative study informed by the PEN-3 cultural model. Ethn Health. 2022 Aug 18;27(6):1358–76.

13. Chinenye S, Ogbera AO. Socio-cultural aspects of diabetes mellitus in Nigeria. J Soc Health Diabetes. 2013 Jun;01(1):15–21.

14. Peabody JW, Taguiwalo MM, Robalino DA, Frenk J. Improving the Quality of Care in Developing Countries. In: Jamison DT, Breman JG, Measham AR, Alleyne G, Claeson M, Evans DB, et al., editors. Disease Control Priorities in Developing Countries [Internet]. 2nd ed. Washington (DC): The International Bank for Reconstruction and Development / The World Bank; 2006 [cited 2023 Jun 18]. Available from: http://www.ncbi.nlm.nih.gov/books/NBK11790/

15. Ashu JT, Mwangi J, Subramani S, Kaseje D, Ashuntantang G, Luyckx VA. Challenges to the right to health in sub-Saharan Africa: reflections on inequities in access to dialysis for patients with end-stage kidney failure. Int J Equity Health. 2022 Sep 5;21(1):126.

16. Nam S, Chesla C, Stotts NA, Kroon L, Janson SL. Barriers to diabetes management: Patient and provider factors. Diabetes Res Clin Pract. 2011 Jul 1;93(1):1–9.

17. Tessema ZT, Worku MG, Tesema GA, Alamneh TS, Teshale AB, Yeshaw Y, et al. Determinants of accessing healthcare in Sub-Saharan Africa: a mixed-effect analysis of recent Demographic and Health Surveys from 36 countries. BMJ Open. 2022 Jan 31;12(1):e054397.

18. Bresick G, Christians F, Makwero M, Besigye I, Malope S, Dullie L. Primary health care performance: a scoping review of the current state of measurement in Africa. BMJ Glob Health. 2019 Sep 13;4(Suppl 8):e001496.

19. Levesque JF, Harris MF, Russell G. Patient-centred access to health care: conceptualising access at the interface of health systems and populations. Int J Equity Health. 2013 Mar 11;12:18.

20. Ameh S, Akeem BO, Ochimana C, Oluwasanu AO, Mohamed SF, Okello S, et al. A qualitative inquiry of access to and quality of primary healthcare in seven communities in East and West Africa (SevenCEWA): perspectives of stakeholders, healthcare providers and users. BMC Fam Pract. 2021 Feb 25;22(1):45.

21. Stokes A, Berry KM, Mchiza Z, Parker W ah, Labadarios D, Chola L, et al. Prevalence and unmet need for diabetes care across the care continuum in a national sample of South African adults: Evidence from the SANHANES-1, 2011-2012. PLOS ONE. 2017 Oct 2;12(10):e0184264.

22. Rural-Urban Differences in Diabetes Care and Control in 42 Low- and Middle-Income Countries: A Cross-sectional Study of Nationally Representative Individual-Level Data | Diabetes Care | American Diabetes Association [Internet]. [cited 2023 Jun 19]. Available from: https://diabetesjournals.org/care/article/45/9/1961/147155/Rural-Urban-Differences-in-Diabetes-Care-and

23. de-Graft Aikins A, Kushitor M, Koram K, Gyamfi S, Ogedegbe G. Chronic non-communicable diseases and the challenge of universal health coverage: insights from community-based cardiovascular disease research in urban poor communities in Accra, Ghana. BMC Public Health. 2014 Jun 20;14(2):S3.

24. Quality of care [Internet]. [cited 2023 Jun 18]. Available from: https://www.who.int/health-topics/quality-of-care

25. Adu MD, Malabu UH, Malau-Aduli AEO, Malau-Aduli BS. Enablers and barriers to effective diabetes self-management: A multi-national investigation. PLoS ONE. 2019 Jun 5;14(6):e0217771.

26. Powers MA, Bardsley JK, Cypress M, Funnell MM, Harms D, Hess-Fischl A, et al. Diabetes Self-management Education and Support in Adults With Type 2 Diabetes: A Consensus Report of the American Diabetes Association, the Association of Diabetes Care & Education Specialists, the Academy of Nutrition and Dietetics, the American Academy of Family Physicians, the American Academy of PAs, the American Association of Nurse Practitioners, and the American Pharmacists Association. Diabetes Care. 2020 Jun 11;43(7):1636–49.

27. Kaplan HC, Provost LP, Froehle CM, Margolis PA. The Model for Understanding Success in Quality (MUSIQ): building a theory of context in healthcare quality improvement. BMJ Qual Saf. 2012 Jan 1;21(1):13–20.

28. Greenhalgh T, Robert G, Macfarlane F, Bate P, Kyriakidou O. Diffusion of Innovations in Service Organizations: Systematic Review and Recommendations. Milbank Q. 2004 Dec;82(4):581–629.

29. Mirzoev T, Das M, Ebenso B, Uzochukwu B, Rawat B, Blok L, et al. Contextual influences on the role of evidence in health policy development: what can we learn from six policies in India and Nigeria? Evid Policy. 2017 Jan;13(1):59–79.

30. Arksey H, O'Malley L. Scoping studies: towards a methodological framework. Int J Soc Res Methodol. 2005 Feb 1;8(1):19–32.

31. Levac D, Colquhoun H, O'Brien KK. Scoping studies: advancing the methodology. Implement Sci. 2010 Sep 20;5(1):69.

32. 11.2.6 Source of evidence selection - JBI Manual for Evidence Synthesis - JBI Global Wiki [Internet]. [cited 2023 May 7]. Available from: https://jbi-global-wiki.refined.site/space/MANUAL/4688854/11.2.6+Source+of+evidence+selection

33. 11.2.7 Data extraction - JBI Manual for Evidence Synthesis - JBI Global Wiki [Internet]. [cited 2023 Apr 1]. Available from: https://jbi-global-wiki.refined.site/space/MANUAL/4687700

34. Tricco AC, Lillie E, Zarin W, O'Brien KK, Colquhoun H, Levac D, et al. PRISMA Extension for Scoping Reviews (PRISMA-ScR): Checklist and Explanation. Ann Intern Med. 2018 Oct 2;169(7):467–73.

35. Page MJ, McKenzie JE, Bossuyt PM, Boutron I, Hoffmann TC, Mulrow CD, et al. The PRISMA 2020 statement: an updated guideline for reporting systematic reviews. BMJ. 2021 Mar 29;372:n71.

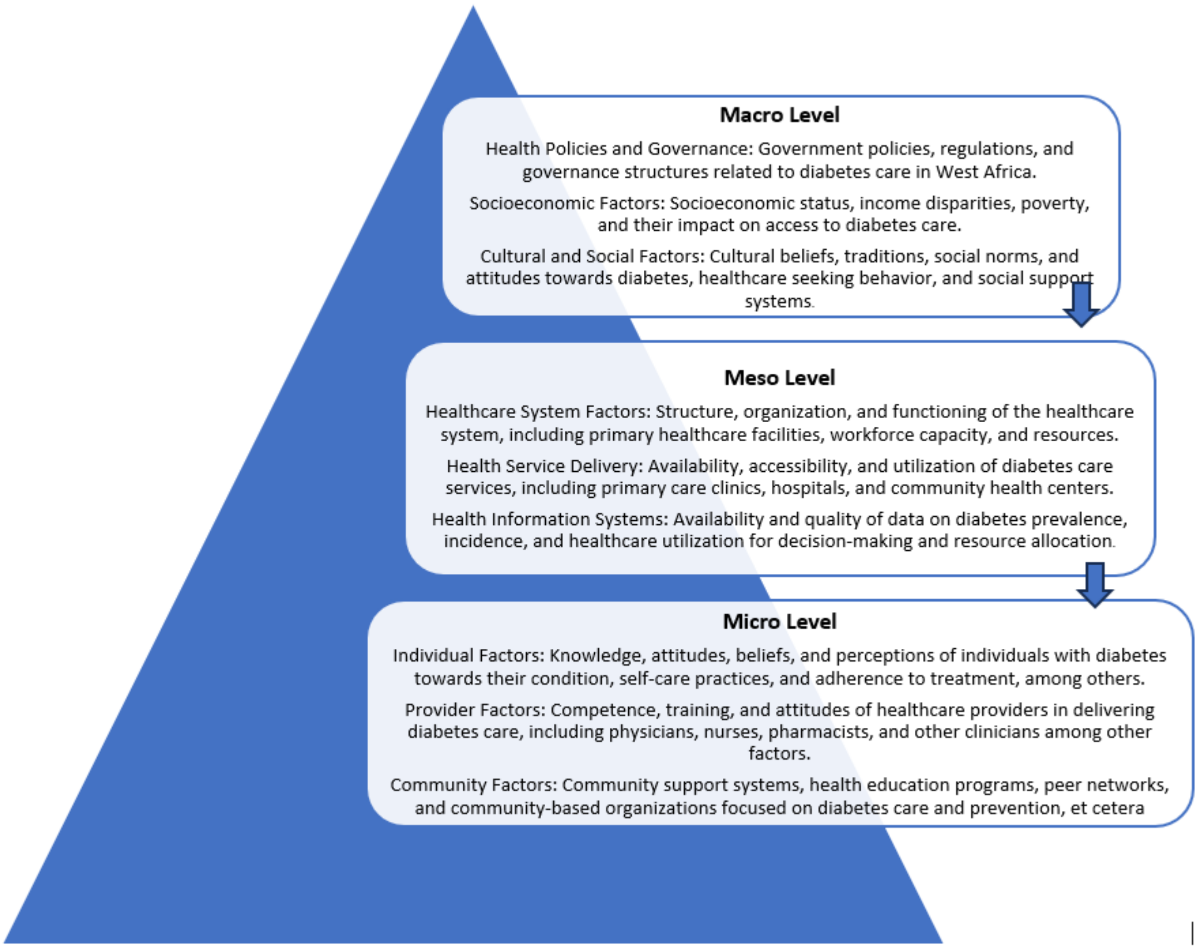
Macro Level
Health Policies and Governance: Government policies, regulations, and governance structures related to diabetes care in West Africa.
Socioeconomic Factors: Socioeconomic status, income disparities, poverty, and their impact on access to diabetes care.
Cultural and Social Factors: Cultural beliefs, traditions, social norms, and attitudes towards diabetes, healthcare seeking behavior, and social support systems.
Meso Level
Healthcare System Factors: Structure, organization, and functioning of the healthcare system, including primary healthcare facilities, workforce capacity, and resources.
Health Service Delivery: Availability, accessibility, and utilization of diabetes care services, including primary care clinics, hospitals, and community health centers.
Health Information Systems: Availability and quality of data on diabetes prevalence, incidence, and healthcare utilization for decision-making and resource allocation.
Micro Level
Individual Factors: Knowledge, attitudes, beliefs, and perceptions of individuals with diabetes towards their condition, self-care practices, and adherence to treatment, among others.
Provider Factors: Competence, training, and attitudes of healthcare providers in delivering diabetes care, including physicians, nurses, pharmacists, and other clinicians among other factors.
Community Factors: Community support systems, health education programs, peer networks, and community-based organizations focused on diabetes care and prevention, et cetera

Figure
Click here to access/download;Figure;Fig.2.docx

PRISMA 2020 flow diagram for new systematic reviews which included searches of databases, registers and other sources

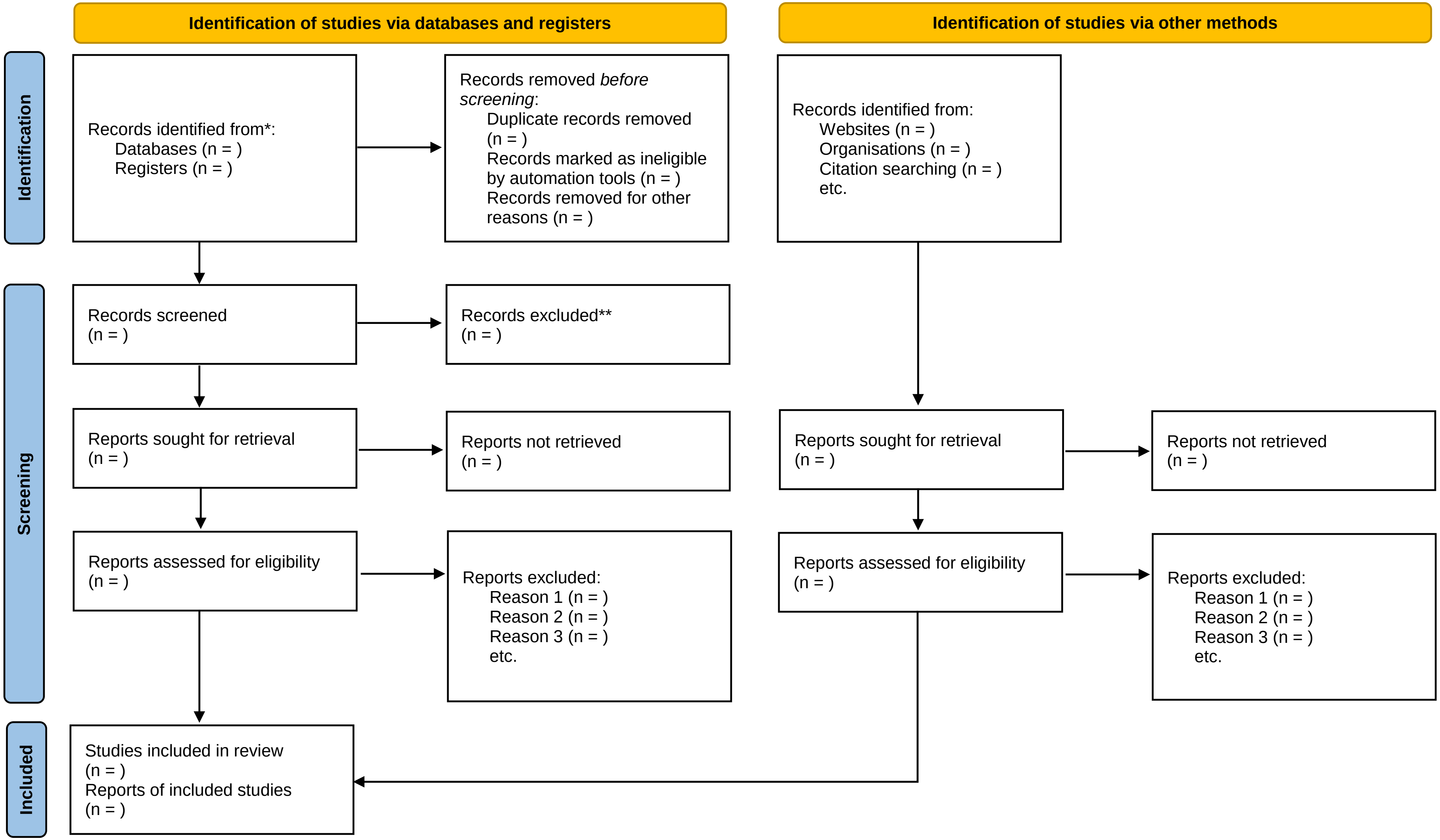

*Consider, if feasible to do so, reporting the number of records identified from each database or register searched (rather than the total number across all databases/registers).
**If automation tools were used, indicate how many records were excluded by a human and how many were excluded by automation tools.

From: Page MJ, McKenzie JE, Bossuyt PM, Boutron I, Hoffmann TC, Mulrow CD, et al. The PRISMA 2020 statement: an updated guideline for reporting systematic reviews. BMJ 2021;372:n71. doi: 10.1136/bmj.n71. For more information, visit: http://www.prisma-statement.org/

Classification: Protected A

Click here to access/download
Supporting Information
S1_Table.docx

Click here to access/download
Supporting Information
S2_Table.docx

Click here to access/download
Supporting Information
S3_Table.docx

Click here to access/download
Supporting Information
S4_Figure.docx

Click here to access/download
Supporting Information
S5_Table.docx

Click here to access/download
Supporting Information
S6_Table.doc
